# Decentralised rare disease care for children living in remote areas: the Pilbara Hub Rare Care model in Western Australia

**DOI:** 10.1016/j.lanwpc.2026.102006

**Published:** 2026-10-24

**Authors:** Bradley MacDonald, Maddison Burmaz, Mathew Bacon, Shelley Bolton, Sue Baker, Kaila Stevens, Amanda Newell, Megan Baxter, Anna Thetford, Gareth Baynam

**Affiliations:** a Rare Care Centre, Perth Children's Hospital, Western Australia, Australia; b Rare Care Pilbara Hub, Western Australia Country Health Service, Western Australia, Australia; c Curtin Medical School and Curtin Health Innovation Research Institute, Curtin University, Western Australia, Australia; d Undiagnosed Diseases Program -WA, Genetic Services of WA, King Edward Memorial Hospital, Western Australia, Australia; e Western Australian Register of Developmental Anomalies, King Edward Memorial Hospital, Western Australia, Australia; f Cardiovascular Epidemiology Research Centre, School of Population and Global Health, University of Western Australia, Crawley, Western Australia, Australia; g Rural Clinical School of Western Australia/School of Medicine, University of Western Australia, Crawley, Western Australia, Australia; h Wesfarmers Centre of Vaccines and Infectious Diseases, The Kids Research Institute Australia, Nedlands, Western Australia, Australia; i Department of Genetics, Guys' and St Thomas' NHS Foundation Trust, London; j Centre for Human Genetics, Oxford University, Oxford; k School of Medicine and Dentistry, Griffith University, Gold Coast, Australia

Children with Rare Diseases (RD) in rural and remote Australia experience profound inequities in access to coordinated multidisciplinary care.^1^, ^2^ In the Pilbara region of Western Australia (WA), a vast region spanning over 500,000 square kilometres and located over 1500 km from tertiary paediatric services, children with RD face barrier to diagnosis, specialist review and continuity of care. An estimated 2000 children in the Pilbara are living with RD but service delivery remains constrained by workforce shortages, fragmented care pathways and a dependence on metropolitan models of care with travel required for necessary care.^3^ These challenges disproportionately influence Aboriginal children and families in dispersed communities, compounding inequity.

Our current metropolitan-centric RD care models are somewhat poorly adapted to the realities of the Pilbara, remaining limited in providing culturally relevant, local and sustainable support for children with complex healthcare needs. A metropolitan based Rare Care Centre (RCC), commenced in 2022, provides cross-sector care coordination for children and families living with RD across WA^4^; with an estimated 63,000 WA children with RD.^5^ For many regional patients, care is limited to telehealth reviews, with limited local perspective or continuity of care.^6^ As a result, there is often reduced access for children with complex lifelong conditions to multidisciplinary support. The RCC metropolitan model remains restricted in resolving persistent inequities in rural and remote settings^7^ with the consequence being RD families with risk of fragmented management, financial burden and health inequity.

The Pilbara Rare Care Hub, a concept developed over the past few years and commenced in 2026, decentralises expert multidisciplinary RD care through integration with local workforce and regionally adapted service delivery.^3^ Flexible referral pathways and a rotating outreach model improve care access to specialist care whilst enabling continuity across geographically dispersed communities. Unlike traditional outreach services, the Pilbara Rare Care Hub embeds local clinicians, educators, disability support providers and social services within the model to strengthen local capability and reduce fragmentation between sectors. Shared governance with regional health services enables adaptation to local priorities and workforce capabilities, while partnerships with Aboriginal Community Controlled Health Organisations and Aboriginal health care workers support culturally responsive care. Similar to the metropolitan model, the early implementation of the model in 2026, has begun to demonstrates high consumer acceptability, improved continuity of care closer to home and with hope that is will yield a return on investment comparable to the RCC.^8^, ^9^ Importantly, the Pilbara Rare Care Hub represents a scalable framework for decentralised RD care in geographically challenging settings where metropolitan tertiary models are somewhat restricted in delivering equitable access.

The Pilbara Rare Care Hub introduces elements of regional service innovation; designed to help develop policy and frameworks to assist in decentralised RD care relevant for dispersed populations globally. As it evolves, evaluation remains critical to determine how the model influences equity, continuity of care workforce sustainability and health system outcomes over the long term. Through implementation science approaches we can identify the transferable components of the model for diverse regional settings, including those with limited workforce capacity and substantial Indigenous health inequities. As healthcare systems across similar regions prioritise equitable RD care access, scalable and culturally responsive partnership models such as the Pilbara Rare Care Hub may offer a blueprint towards management of geographic disparities in lifelong chronic conditions.

Across the Western Pacific, children with RD in dispersed, underserviced communities continue to face substantial inequity to specialist care. The Pilbara Rare Care Hub offers a template for how decentralised, locally embedded models can begin to close this gap with the building of local capacity while retaining multidisciplinary input. Through planned evaluation and regional reporting, the models impact on equity, continuity of care and workforce sustainability will be tracked. As health systems in the Western Pacific seek strategies to reduce geographic inequity, the Pilbara model may offer a scalable, transferable approach to decentralised RD care in comparable settings.

## Declaration of interests

The authors declare no competing interests.
